# Detection of the enterotoxigenic genes (*sei,sej*) in *Staphylococcus aureus* isolates from bovine mastitis milk in the West Azerbaijan of Iran

**DOI:** 10.1007/s00580-012-1460-3

**Published:** 2012-03-15

**Authors:** Malahat Ahmady, Sahar Kazemi

**Affiliations:** 1 Department of Microbiology, Faculty of Veterinary Medicine, Urmia University, P.O. Box 1177, Urmia, Iran; 2 Veterinary Medicine, Urmia University, P.O. Box 1177, Urmia, Iran

**Keywords:** *S. aureus*, Bovine mastitis, Enterotoxin, PCR

## Abstract

*Staphylococcus aureus* is a major causative pathogen of clinical and subclinical mastitis of dairy domestic ruminants. This organism produces a variety of extracellular toxins and virulence factors such as enterotoxin SEI and SEJ that contribute to its pathogenic potential. In this study 25 *S. aureus* isolates obtained from four dairy herds of Urmia region which is located in West Azerbaijan province in Iran. The tested isolates were identified on the basis of the cultural and biochemical properties, as well as amplification of the *aroA* gene which is specific for *S. aureus*. All isolates were also analyzed for the presence of the SEI (*sei*) and SEJ (*sej*) encoding genes using polymerase chain reaction (PCR). Seven positive isolates were detected for *sei*, but *sej* gene was not detected in any of the total number of 25 isolates. The present study revealed that the PCR amplification of the *aroA* gene could be used as a powerful tool for identification of *S. aureus* from the cases of bovine mastitis. Results of the present study also showed that the strains of *S. aureus* which cause mastitis can potentially produce enterotoxin SEI. Overall, our results suggest that it is of special importance to follow the presence of enterotoxin-producing *S. aureus* in other dairy products, especially for protecting the consumers from staphylococcal food poisoning.

## Introduction

*Staphylococcus aureus* is the cause of a widespread spectrum of infections in humans and different animal species. It has been isolated frequently from bovine mastitis (Zschöck et al. 2005). Major costs due to mastitis are associated with reduced milk production, reduced value of animal, discarded milk, early replacement, reduced sale, additional veterinary service and labor (Middleton et al. 2002). In addition, enterotoxigenic *S. aureus* is one of the major pathogens causing food poisoning worldwide (Dinges et al. 2000). Milk is a good substrate for *S. aureus* growth and enterotoxin production which retains their biological activity even after pasteurization (Asoa et al. 2003). Staphylococcal enterotoxins (SEs) have been divided into five major serological types (SEA, SEB, SEC, SED, and SEE) on the basis of their antigenic properties (Bayles and Iandolo 1989; Betley and Mekalanos 1988; Bohach and Schlievert 1987; Couch et al. 1988; Letertre et al. 2003). Recently, new types of SE (SEG, SHE, SEI, SEJ, SEK, SEL, SEM, SEN, and SEO) have been identified (Abe et al. 2000; Fitzgerald et al. 2001; Jarraud et al. 2001; Munson et al. 1998; Orwin et al. 2001; Ren et al. 1994; Zhang et al. 1998; Omoe et al. 2005). However, the role of newly identified enterotoxins in food poisoning is not fully clarified, and the development of methods for the detection of these novel *se* toxin genes is of critical importance for food poisoning investigations. Various methods have been developed for detecting the production of enterotoxins from *S. aureus*. SEs can be routinely detected by immunoassay, e.g., enzyme-linked immunosorbent assay (ELISA), immunodiffusion, radioimmunoassay, and latex agglutination, but these are usually limited to SEA, SEB, SEC, SED, and SEE. In addition, the detection limit and specificity of these methods always depend on having detectable amounts of toxins and may vary significantly according to reagent purity. Since DNA sequence information is available for all described SEs, DNA–DNA hybridization and PCR now offer alternative methods for detection of these genes (Gilligan et al. 2000; Johnson et al. 1991; McLauchlin et al. 2000; Omoe et al. 2002). Because of the importance of the enterotoxins on public health and great geographical variation in the distribution of enterotoxigenic strains, in this study, the distribution of the genes that encode SEJ and SEI was analyzed by PCR in *S. aureus* isolated from bovine mastitis.

## Materials and methods

### Sampling, isolation, and identification of *S. aureus*

A total of 160 milk samples were collected according to the recommendations of the National Mastitis Council (1987) methods from four Holstein dairy herds in West Azerbaijan in Iran. Cows were chosen based on clinical and subclinical mastitis, clinical mastitis recognized by observation and palpation of the udder, presence of clots in the milk, and inflammation in the infected quarter. Cows with subclinical mastitis have mammary gland without clinical abnormalities and apparently normal milk, so they checked by bacteriological tests and with California Mastitis Test.

Teats were thoroughly washed, dried with clean towel, and sprayed with 70 % ethanol. The first few jets of milk were discarded, and 10 ml of milk samples from each quarter was collected in a sterile McCartney bottle. All samples were kept at 4°C and transported immediately to the lab for latter examination.

Each milk sample (100 μl) was cultured on the surface of mannitol salt agar (Merck, Germany) and was incubated at 37°C for 24–48 h. Colonies suspected as *S. aureus* were selected and transferred to 5 % sheep blood agar (Difco). Gram stain, culture characteristics, and coagulase test using fresh rabbit plasma (tube method) were used for the presumptive identification of all isolates (National Mastitis Council 1999). A total of 25 isolates of *S. aureus* were isolated from the bovine mastitis milk samples.

### DNA extraction

*S. aureus* DNA was prepared from overnight cultures in 10 ml of brain infusion broth (Merck, Germany) by DNA purification kit (Fermentas, Germany) according to the manufacturer’s recommendation.

### Amplification of the *aroA* gene

*S. aureus* species confirmation was also performed by polymerase chain reaction amplification of the *aroA* gene, as described by Marcos et al. (1999) with some modifications. The amplification of *aroA* gene has done with a pair of primer and thermal profile which is shown in Table 1.

**Table 1:** Oligonucleotide primers and PCR programs for amplification of *aroA* gene

Primers	Sequence(5′–3′)	PCR program^a^	Size (bp)	Reference
*aroA*		1	1,153	Letertre et al. (2003)
FA1	AAG GGC GAA ATA GAA GTG CCG			
RA2	CAC AAG CAA CTG CAA GCA T			

1^a^Thirty-two times (92°C, 1 min; 63°C, 1 min; 72°C, 1.5 min)

### PCR testing for genes *sei,sej*

The PCR amplification was carried out in 0.5-ml tubes in the reaction final volume of 50 μl. The PCR mixture consisted of 25 μl of 2× master mix (0.04 U/μl Taq DNA polymerase reaction buffer, 3 mM MgCl_2_ 0.4 mM of each dNTP), 0.8 μM of each primer, and 4 μl of extracted DNA. The tubes were subjected to thermal cycling (Corbett Research CP2-2003, Australia) with the program shown in Table 2.

**Table 2:** Oligonucleotide primers and PCR programs for amplification of the genes encoding staphylococcal toxins SEI and SEJ

Primers	Sequence(5′–3′)	PCR program^a^	Size (bp)	Reference
*sei*		2	576	Jarraud et al. (1999)
SEI-1	CTC AAG GTG ATA TTG GTG TAGG			
SEI-2	AAA AAA CTT ACA GGC AGT CCA TCT C			
*sej*		3	146	Monday and Bohach (1999)
SEJ-1	CAT CAG AAC TGT TGT TCC GCT AG			
SEJ-2	CTG AAT TTT ACC ATC AAA GGT AC			

2^a^Thirty times (94°C, 2 min; 55°C, 2 min; 72°C, 1 min); 3^a^ 30 times (94°C, 1 min; 62°C, 1 min; 72°C, 1 min)

The amplified PCR products were visualized simultaneously by standard gel electrophoresis in a 1.2 % agarose gel (Sigma, Germany), stained with ethidium bromide (0.5 μg/ml). The gels were photographed under ultraviolet light using the Bio profile system (BTS-20, Japan). Molecular size markers (100-bp and 1-kb DNA ladder; Fermentas, Germany) were included in each agarose gel.

## Results

**Fig. 1:**
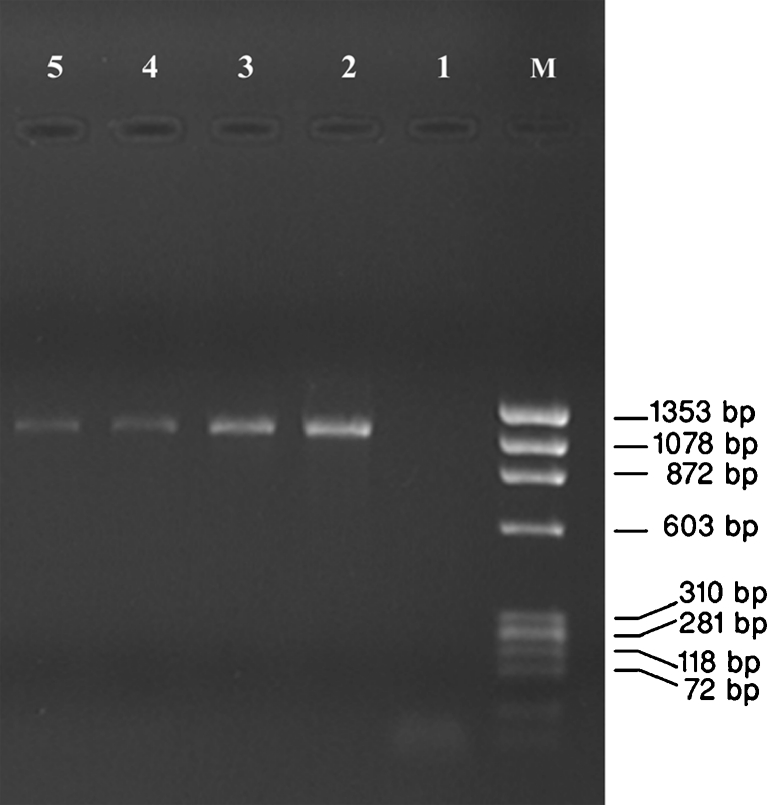
Agarose gel electrophoresis of amplification of *aroA* gene. *Lane M* marker ФX174 DNA/HaeIII; *1* negative control (sterile water was added instead of DNA); *2* positive control (*S. aureus* ATCC29213); *3*, *4*, and *5* PCR product with expected size 1,153 bp

**Fig. 2:**
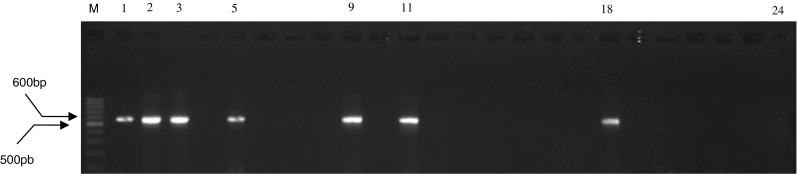
Agarose gel electrophoresis of amplification of *sei* gene. *Lane M* marker 100 bp (Fermentas, Germany); *1*, *2*, *3*, *5*, *9*, *11*, and *18* PCR product with expected size 576 bp; *24* negative control (sterile water was added instead of DNA)

From 160 milk samples examined bacteriologically, 25 *S. aureus* were isolated and confirmed using detection of *aroA* gene with expected size of approximately 1,153 bp which is specific for *S. aureus* that is shown in Fig. 1. All *S. aureus* isolates were also analyzed for the presence of the SEI (*sei*) and SEJ (*sej*) encoding genes using PCR. Seven positive isolates were detected for sei that is shown in Fig. 2, but *sej* gene was not detected in any of the total number of 25 isolates (no figure).

## Discussion

*S. aureus* is involved in intramammary infections in bovine causing economic losses and milk safety problems (Taverna et al. 2007). Mastitis control is a complex problem for which there are no simple solutions. Bacteriological study of mastitic milk samples was carried out on 160 milk samples, and the results revealed that *S. aureus* was present in 25 samples with the percentage of 15.65 %.The identification of the 25 *S. aureus* isolates in the present study was carried out by conventional methods and by PCR technology. The latter uses oligonucleotide primers targeted to *aroA* gene. This gene allows a rapid identification of *S. aureus* with high sensitivity and specificity (Saei et al. 2010; Marcos et al. 1999; El-Huneidi et al. 2006). The amplification of the *aroA* gene revealed an amplification with a size of 1,153 bp for all *S. aureus* isolates investigated. *S. aureus* is characterized by many virulence factors and by large variations in the presence of these genes. Such different gene patterns should be considered when assessing their specific role in the development of infections(Peacock et al. 2002; Piccinini et al. 2009; Zecconi et al. 2006). Major virulence factors of *S. aureus* organism include enterotoxins (SEs) that cause both food poisoning and toxic shock syndrome (Orwin et al. 2003). For a long time, among the *S. aureus* enterotoxin types, only SEA–SEE have been linked to bovine, ovine, or caprine strains (Kenny et al. 1993; Matsunaga et al. 1993; Zschöck et al. 2000, 2005). The present study shows the occurrence of recently described enterotoxin genes *sei* and *sej* in randomly selected bovine mastitis isolates of *S. aureus* by PCR. Although it is possible to detect the gene products immunologically using a variety of ELISA and radioimmunoassays (Johnson et al. 1991), these tests have variable sensitivities and depend on adequate gene expression for reliability and reproducibility. Toxigenic strains of *S. aureus* with low levels of excreted toxin(s) or cross-reactive antigens could therefore be easily misidentified by immunologic methods. In our study the prevalence of sei gene was 28 %. But different values were reported by others. Rall et al. (2008) detected 10 (17.54 %) positive strains out of 57 samples of *S. aureus* which were obtained from raw and pasteurized milk in Brazil, and Zschöck et al. (2005) have found 61 % positive strains for *sei* from *S. aureus* isolates of bovine mastitic milk samples in Germany. No *sej* gene was detected from the isolates which were examined in the present study. This finding was in contrast with Rall et al. (2008) who detected three (7.7 %) positive strains, and Zschöck et al. (2005) found 37.7 % positive strains for *sej*. But Jorgensen et al. (2005) reported that the gene *sej* has been found only in *S. aureus* associated with *seg* and *sei*. The occurrence of multiple toxin genes in *S. aureus* is considered rare which can explain our negative result for the *sej* gene.

Results of these studies revealed that staphylococcal enterotoxic genes have a broad distribution worldwide and also showed the differences in the geographical distribution of these genes. The pattern for *S. aureus* intramammary infection is often different from herd to herd, and these patterns could be related to strain differences (Joo et al. 2001; Van Leeuwen et al. 2005; Zecconi et al. 2005; Piccinini et al. 2010). These differences have been also related to geographical and to host and tissue-related characteristics (Gilot and Van Leeuwen 2004; Van Leeuwen et al. 2005). In contrast (De centorbi et al. 1988; Kenny et al. 1993; Matsunaga et al. 1993; Takeuchi et al. 1998; Larsen et al. 2002; Morandi et al. 2007; Aarestrup et al. 1995) studies of the presence of sag’s (superantigens) *S. aureus* isolated from Danish cases of clinical and subclinical bovine mastitis showed that Danish bovine *S. aureus* generally did not carry the genes for known sags (it is necessary to mentioned that the presence of *seg,sei* and *sej* gene were not examined). The results of these studies demonstrated the marked and highly significant geographical variation in the presence of sags. On this background, we can result that the superantigens investigated in these study do not play an important role in the pathogenesis of bovine *S. aureus* mastitis. If the sags participate in the pathogenesis of bovine *S. aureus* mastitis to any significance, the contagiousness, the clinical manifestations, or the course of infection should show geographical variation in congruence with the variation of the presence of the exotoxin genes in bovine *S. aureus* isolates (Larsen et al. 2002).

We should consider that when a strain has an enterotoxigenic gene, it can potentially produce enterotoxins. Jorgensen et al. (2005) reported that the gene *sej* has been found only in *S. aureus* associated with *seg* and *sei*. The occurrence of multiple toxin genes in *S. aureus* is considered rare which can explain our negative result for the *sej* gene. Many other conditions are necessary. *S. aureus* produce enterotoxins throughout the logarithmic phase of growth or during the transition from the exponential to the stationary phase (Betly and Mekalanos 1988; Otero et al. 1990). Also it has been reported that the production of SEs may depend on the host environment and may play a role in the adaptation of *S. aureus* to the host (Banks et al. 2003). Jorgensen et al. (2005) reported that the gene *sej* has been found only in *S. aureus* associated with *seg* and *sei*. The occurrence of multiple toxin genes in *S. aureus* is considered rare which can explain our negative result for *sej* gene.

Finally the importance of toxin formation by *S. aureus* for udder pathogenesis remains unclear. According to Akineden et al. (2001), the superantigenic toxins seem to induce immunosuppression in dairy animals.
